# Variable Energy Availability Exposure and Heterogeneous Physiological Responses to a Prescribed Low Energy Availability Intervention in Free-Living Male Endurance Athletes: A Pilot Case-Series

**DOI:** 10.3390/sports14100446

**Published:** 2026-10-09

**Authors:** Fabricio Forchino, Carly J. Brade, Deborah A. Kerr, Dale W. Chapman

**Affiliations:** 1 School of Allied Health, Curtin University, Bentley, WA 6102, Australia; fabricio.forchino@postgrad.curtin.edu.au (F.F.); carly.brade@curtin.edu.au (C.J.B.); 2 School of Population Health, Curtin University, Bentley, WA 6102, Australia; d.kerr@curtin.edu.au

**Keywords:** energy intake, exercise physiology, applied sport science, exercise energy expenditure, performance, athlete monitoring

## Abstract

Low energy availability (LEA) is commonly studied under tightly controlled laboratory conditions. However, its induction and monitoring in free-living athletes remain poorly understood, partly due to challenges in measuring energy intake (EI), exercise energy expenditure (EEE) and daily behavioural variability. This study examined whether a prescribed LEA intervention leads to a consistent energy deficit and described the variability in energy availability (EA) and associated physiological and performance responses. In this pilot case series with a repeated-measures control-intervention structure, trained male endurance athletes (*n* = 3) completed a 14-day intervention targeting LEA (~20 kcal (~84 kJ)·kg fat-free mass (FFM)^−1^·day^−1^) by increasing EEE while maintaining habitual EI. EA was estimated daily using image-based dietary assessment, heart rate-derived EEE and dual-energy X-ray absorptiometry-derived FFM; resting metabolic rate (RMR), performance and well-being were assessed pre- and post-intervention. Despite the target, achieved EA varied substantially within and between participants (day-to-day SD 7–20 kcal·kg FFM^−1^·day^−1^), reflecting the magnitude and distribution of EEE rather than a uniform deficiency. Responses were heterogeneous: RMR showed no consistent direction, haemoglobin decreased modestly in all athletes, body-composition changes were small, and performance ranged from decline to improvement. Mean EA interpreted in isolation may obscure intra-individual variability, indicating a need for methods that capture it in free-living research.

## 1. Introduction

Athletes engaging in high training loads are frequently exposed to periods of low energy availability (LEA), a state in which dietary energy intake (EI) is insufficient to support both exercise demands and optimal physiological function [1,2]. Energy availability (EA), defined as the difference between EI and exercise energy expenditure (EEE) relative to fat-free mass (FFM), is commonly used to quantify this state [3]. Thresholds below 30 kcal (125 kJ)·kg FFM^−1^·day^−1^ are proposed as potentially detrimental; however, these thresholds originated primarily from tightly controlled laboratory studies in small samples of non-athletic women, limiting their generalisability to athletic populations [4,5,6].

In female athletes, LEA is well documented and associated with hypothalamic suppression, menstrual dysfunction, impaired bone health, increased injury risk, and reduced performance [1,6,7]. Although evidence in male athletes is limited and less consistent, LEA has also been linked to endocrine disruption, reduced testosterone and leptin concentrations, increased susceptibility to illness, and lower bone mineral density [2,8,9]. Emerging evidence suggests that male athletes may be more tolerant to lower EA levels than female athletes; however, the physiological consequences of sustained LEA in men remain unknown [10,11]. In exercising men, 4 days at 15 kcal·kg FFM^−1^·day^−1^ reduced leptin and insulin without changes in testosterone, triiodothyronine or insulin-like growth factor 1 [10], suggesting that several endocrine markers which respond in women are more robust to short LEA exposures in men. In trained male endurance athletes, 14 days of exercise-induced LEA at approximately 22 kcal·kg FFM^−1^·day^−1^ lowered haemoglobin concentration, reduced explosive power and worsened self-reported well-being, whereas measured resting energy expenditure did not change [12]. A further stepwise reduction to approximately 17 kcal·kg FFM^−1^·day^−1^ extended these changes to body composition and further performance measures [13]. Consistent with these observations, reduced resting metabolic rate (RMR) or RMR ratio, a decline in haemoglobin or iron status, sleep disturbance and psychological symptoms are listed as potential indicators of Relative Energy Deficiency in Sport (REDs), and a significant within-athlete change from baseline in any indicator is regarded as a sign [14]. Outcomes that have changed within 14 days in men, that are listed as REDs indicators and that can be measured outside the laboratory therefore offer the most defensible basis for detecting early responses to LEA in free-living male endurance athletes.

In applied sport settings, assessing EA is methodologically complex. Accurate quantification requires precise measurement of EI, EEE, and FFM, each of which is subject to considerable measurement error [15,16]. Dietary assessment in athletes is particularly challenging due to high and variable EI, frequent eating, and the use of sports foods and supplements, all of which contribute to misreporting and day-to-day variability [17,18]. Similarly, estimation of EEE outside laboratory conditions relies on indirect approaches such as heart rate (HR) monitoring, power output, wearable devices, and activity logs, which introduce systematic bias compared with gold-standard methods such as doubly labelled water [19,20]. Furthermore, variability in body composition assessment methods contributes to inconsistent EA estimates across studies [15,21]. These methodological challenges are compounded in free-living athletes, where both EI and exercise behaviour fluctuate daily. Unlike laboratory-based studies, free-living environments do not allow strict control over dietary intake, exercise, or daily activity patterns. As a result of these challenges, achieving and maintaining a target EA in a free-living athlete environment is hypothesised to be difficult, and the extent to which prescribed interventions translate into actual changes in EA remains unclear.

Beyond the methodological quantification challenges, the duration of LEA exposure represents an additional source of variability. Short-term laboratory studies of less than 7 days exposure may not capture the cumulative effects of LEA observed in athletes over longer periods, and physiological markers are reported to respond differently depending on both the severity and duration of energy deficiency [20,22]. While some markers respond within days, others require prolonged exposure, complicating the interpretation and translation of laboratory findings to applied sporting environments. Given these challenges, there is a need for studies that not only examine the physiological and performance consequences of reduced EA but also evaluate the feasibility of inducing and monitoring LEA under free-living conditions using contemporary assessment approaches.

Therefore, the primary aim of this pilot case series study was to characterise the extent to which a prescribed reduction in EA to approximately 20 kcal·kg FFM^−1^·day^−1^ could be achieved and sustained under free-living conditions in male endurance athletes, by quantifying the difference between the prescribed target and the achieved daily EA and describing its within- and between-participant variability across a 14-day intervention. A secondary aim was to describe physiological and exercise performance responses to the intervention, interpreted in the context of individual EA exposure profiles. We anticipated that achieved EA would vary substantially both within and between participants, and that physiological and performance responses would reflect this heterogeneity in exposure rather than a uniform LEA effect. This approach is consistent with methodological guidance for single-subject and case-based designs in applied sport science, which recommends this design specifically when inter-individual differences in response are expected to be large relative to the average effect [23]. It is reinforced by practical constraints: the intensive per-participant measurement required to estimate EA daily, under free-living conditions, combined with the logistical demands of a 14-day prescribed intervention, constrains feasible sample sizes and controlled EA research in trained athletes has consistently relied on small cohorts for this reason [13,24]. Case-based designs have established precedent in this field and preserve the participant-specific detail necessary to characterise heterogeneous exposure and responses [25,26], and recent methodological guidance confirms that a rigorously structured case series can generate original, defensible insight of this kind [27]. This approach therefore provides novel insight into the ecological validity of current LEA frameworks by evaluating them against real, rather than averaged, athlete exposure profiles.

## 2. Materials and Methods

### 2.1. Study Design

This pilot study employed a case series design with a repeated-measures, control–intervention structure, in which each athlete served as their own control. The purpose was to characterise the physiological and performance responses to a 14-day intervention aimed at reducing EA in free-living male endurance athletes. The protocol spanned 23 days and consisted of baseline testing (T0), a habitual monitoring period, pre-intervention testing (T1), development of an individualised exercise prescription, a 14-day intervention, and post-intervention testing (T2).

Importantly, rather than assuming that a fixed EA level could be achieved under free-living conditions, the intervention was designed to attempt to reduce EA to approximately 20 kcal·kg FFM^−1^·day^−1^. This value was not a physiological threshold but an experimentally prescribed target. The 30 kcal·kg FFM^−1^·day^−1^ threshold derives from short laboratory studies of sedentary women [4,5,22]; no equivalent threshold has been established in men, in whom the range at which REDs-related effects appear seems lower [14], whilst 14-day exercise-induced reductions to approximately 22 and 17 kcal·kg FFM^−1^·day^−1^ have produced measurable changes in male endurance athletes [12,13]. A target of 20 was therefore set clearly below 30 and within the previously reported effective range. Accordingly, EA was treated as a variable exposure reflecting both behavioural compliance and measurement variability.

### 2.2. Participants

Four male endurance athletes were recruited via social media, local sporting clubs, and word of mouth. Participants were required to train a minimum of three times per week and to compete at Tier 2 (Trained/Developmental) or above within the participant classification framework of McKay et al. [28], which ranks athletes from Tier 0 (sedentary) to Tier 5 (world class) on the basis of training history and competition standard (Table 1). Eligible participants were aged 18 to 50 years. Eligibility was screened with an online questionnaire (Qualtrics, Provo, UT, USA) in which volunteers self-reported no diagnosed cardiac, respiratory, metabolic or endocrine condition (including diabetes and thyroid disorders), no diagnosed or treated eating disorder, and no cardiovascular symptoms during or after exercise in the preceding 12 months; no clinical examination or biochemical screening was performed. After receiving a verbal description of the study and an opportunity to ask the lead author questions, all participants provided written informed consent prior to participation. Written informed consent for publication of anonymised individual data was also obtained from all participants. The study was approved by the Curtin University Human Research Ethics Committee (HRE2025-0256), and all procedures conformed to the Declaration of Helsinki for research involving human participants.

### 2.3. Experimental Overview

**Figure 1:**
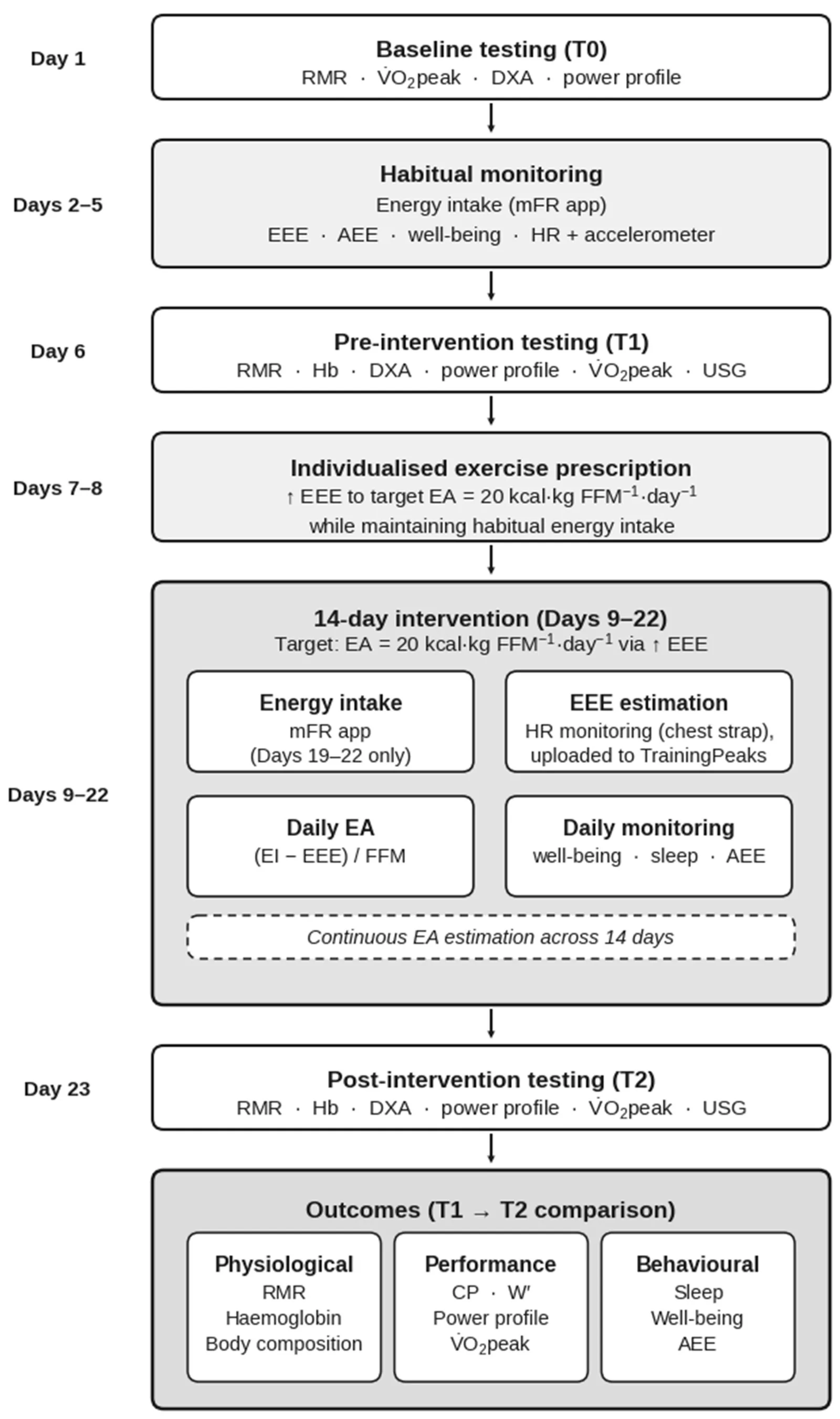
Experimental design and participant flowchart. AEE = activity energy expenditure; CP = critical power; DXA = dual-energy X-ray absorptiometry; EA = energy availability; EEE = exercise energy expenditure; EI = energy intake; FFM = fat-free mass; Hb = haemoglobin; HR = heart rate; mFR = mobile food record; RMR = resting metabolic rate; T0, T1 and T2 = baseline, pre-intervention and post-intervention testing; USG = urine specific gravity; $\dot{V}$O_2_peak = peak oxygen uptake; W′ = work above critical power; within day arrows indicating directional change (days 9–22 an increase) or direction of comparison between T1 and T2 (day 23).

Each athlete was assessed individually and is reported as a separate case because the primary question concerns the extent and variability of achieved EA within and between individuals; a group-level design would average across participants and obscure this variability. We also sought to preserve individual-level detail and to allow the magnitude and temporal pattern of each athlete’s EA exposure and responses to be characterised without aggregation, consistent with prior individual-level analyses of EA in free-living athletes [25,29]. Each athlete completed baseline testing on Day 1 (T0), followed by a 4-day habitual monitoring period (Days 2–5) during which participants followed their usual, self-selected training and diet, to quantify habitual EI, EEE and physical activity under free-living conditions. Pre-intervention testing was conducted on Day 6 under standardised conditions. An individualised exercise prescription was then developed (Days 7–8) based on baseline EI and EEE data, aiming to reduce EA by increasing EEE while maintaining habitual EI. Participants subsequently completed a 14-day intervention (Days 9–22), during which they followed the prescribed training programme. Post-intervention testing was conducted on Day 23 under conditions identical to pre-intervention testing (Figure 1).

### 2.4. Energy Intake Assessment (EI)

EI was assessed using a mobile food record (mFR™) app, an image-based dietary assessment method [30]. Training and instrumentation were provided during the T0 session. Habitual EI was operationalised as the mean daily intake across the 4-day baseline window (Days 2–5) and applied as a constant value per athlete in the calculation of EA; the second 4-day window (Days 19–22) was used to verify that habitual intake had been maintained. Daily EI was therefore not measured on every intervention day. Four-day windows were used because compliance with self-reported intake declines beyond four days [2] and because 3 to 4 days has been reported as sufficient to characterise habitual intake [15]. Dietary recording was scheduled on two 4-day windows (Days 2 to 5 and Days 19 to 22), both specified in the participant information provided before enrolment; no recording was requested on the intervening days, and daily EI was therefore not measured during the intervention. Participants captured images of all foods and beverages before and after consumption, each including a checkerboard fiducial marker supplied by the research team, which provides the reference of known dimensions used for portion-size estimation. Foods were identified and portion sizes estimated from the images by the lead author (F.F.), and the corresponding food codes and weights were entered into dietary analysis software (FoodWorks v.10, Xyris Software, Brisbane, QLD, Australia) with the AusFoods 2019 and AusBrands 2019 food composition databases to estimate daily EI (kcal·day^−1^).

### 2.5. Exercise Energy Expenditure (EEE)

EEE was estimated from HR data continuously recorded during all intervention training sessions using chest-strap HR monitors (Polar H10, Polar Electro Oy, Kempele, Finland) worn by each participant. HR data were uploaded to an online training management platform (TrainingPeaks, Peaksware, Boulder, CO, USA), where gross session EEE (kcal) was estimated using the manufacturer’s proprietary algorithm (Garmin Ltd., Olathe, KS, USA). These estimates were therefore treated as model-derived rather than directly measured values.

EEE was initially treated as gross expenditure, that is, including the resting metabolic component accrued during each intervention training session. Training modality (cycling, running, swimming, or other) and session duration were recorded for all sessions. These values were subsequently used for the estimation of EA.

### 2.6. Energy Availability (EA)

EA was calculated as: EA = (EI − EEE)/FFM and expressed as kcal·kg FFM^−1^·day^−1^.

EA was calculated using baseline EI and EEE data collected during Days 2–5, together with dual-energy X-ray absorptiometry (DXA) derived FFM measured at T1. This pre-intervention EA estimate was used to determine the additional average daily EEE required to reduce EA toward the intervention target of 20 kcal·kg FFM^−1^·day^−1^. The daily EEE required to reach the target was calculated as habitual EI minus the product of the target EA and DXA-derived FFM, and the exercise prescription was individualised to meet this requirement. Expired gases collected during the 240 s effort of the T1 cycling power profile test were used to calculate energy expenditure using the Weir equation [31], from which metabolic power was derived; gross mechanical efficiency was then calculated as the mean mechanical power sustained over that effort divided by metabolic power. Cycling training zones were defined as percentages of critical power following a seven-zone scheme originally described relative to functional threshold power [32], on the basis that critical power provides a more robust anchor for the power-duration relationship than a single-effort threshold estimate such as functional threshold power [33], with corresponding heart rate ranges expressed as percentages of maximal heart rate, and the energy cost of each zone was estimated by converting its mechanical power to metabolic energy using the participant-specific gross efficiency. Time in each zone was then prescribed across the 14 days so that the predicted EEE met the daily requirement.

During the intervention, participants were instructed to maintain their habitual dietary intake while EEE was manipulated through an individualised exercise prescription. All training sessions, whether prescribed cycling or self-selected running, swimming and resistance training, were recorded by the participants’ own devices and uploaded automatically to TrainingPeaks. Completed session EEE was reviewed daily by the lead author and compared with the daily requirement, and the running mean of the difference between completed and required EEE was tracked across the intervention. The pre-specified criterion for adjustment was a deviation of this running mean beyond ±10% of the daily EEE requirement. When this occurred, the prescribed time in each training zone was adjusted so that the mean EA over the 14 days would approach the target. Daily required, prescribed and completed EEE and achieved EA for each participant are provided in Supplementary Table S2. Accordingly, intervention exposure was conceptualised as a dynamically monitored, exercise-driven reduction in EA rather than as a fixed, prescribed deficit. To illustrate the calculation, gross EA on Day 7 of the intervention for Participant 1 (EI = 2200 kcal·day^−1^; FFM = 73.79 kg) was computed as EA = (EI − EEE)/FFM = (2200 − 579)/73.79 = 21.97 kcal·kg FFM^−1^·day^−1^, where EEE = 579 kcal·day^−1^ corresponds to the gross EEE completed on that day. When calculated using net EEE (i.e., subtracting the resting metabolic component accrued during exercise; 473 kcal·day^−1^), EA on the same day was 23.41 kcal·kg FFM^−1^·day^−1^. Because EI was operationalised as the mean daily intake across the 4-day baseline window and applied as a constant value per athlete, daily variation in EA across the intervention period was driven by the magnitude of EEE completed.

To verify whether habitual EI had been maintained, dietary intake was reassessed during the final 4 days of the intervention (Days 19–22). Mean EI over these days was 2178, 2225 and 2863 kcal·day^−1^ for P1, P2 and P3, within 1.3% of the habitual values used (Table 2). Intervention EA was interpreted using participant-specific habitual EI, intervention-period EEE and DXA-derived FFM measured at T1. For primary comparisons, EA was expressed as gross EEE values (i.e., without subtracting the resting energy expenditure that would have occurred during exercise), reflecting the total energetic cost of exercise sessions. This approach was selected to align with the outputs typically available in applied athlete-monitoring platforms and free-living training environments.

In addition, net EEE was calculated as a sensitivity analysis by subtracting the estimated resting energy expenditure accrued during each intervention exercise bout from gross EEE, and corresponding net EA values were derived. This adjustment provided a contextualised interpretation against the original operational definition of EA, which accounts for the energy that would have been expended at rest during the exercise period; failure to make this adjustment may overestimate EEE and underestimate true EA in free-living athletes [3,15]. EA was interpreted as a continuous exposure rather than dichotomised into LEA/non-LEA categories.

### 2.7. Laboratory Assessments

Day 1 (T0) served as a familiarisation and instrumentation session. Participants completed the cycling power profile (CPP) test [34] to acclimatise to the protocol and to record the individual gear ratios subsequently replicated at T1 and T2; the thigh-worn accelerometer (SENS Motion, SENS Innovation ApS, Copenhagen, Denmark) was then fitted, and participants were instructed in the dietary, training, well-being and physical activity monitoring procedures. Pre- and post-intervention testing (Day 6 (T1) and Day 23 (T2)) was conducted in the morning, at a time arranged with each participant, and followed an identical order. Participants arrived having fasted for ≥10 h, having abstained from alcohol and caffeine since the preceding evening, and having refrained from structured exercise since the previous afternoon [35]. No exercise-free day was imposed before either session, so that T2 followed the final intervention day directly. On arrival, completion of the morning well-being questionnaire was verified, urine specific gravity (USG) was assessed, and body mass (BM) was recorded. RMR was then measured, after which participants began consuming a standardised 600 mL carbohydrate–electrolyte drink (Gatorade, Asahi Lifestyle Beverages, Melbourne, VIC, Australia), ingested before and after the body composition assessment which was assessed by DXA. Hydration was reassessed where required and once euhydration was confirmed, capillary haemoglobin was sampled. Finally, the CPP test was performed with water permitted ad libitum, and a light breakfast was provided upon completion.

#### 2.7.1. Hydration Assessment

Hydration status was assessed immediately prior to haemoglobin sampling from a midstream urine sample using a portable refractometer (RS PRO, RS Components, Smithfield, NSW, Australia). This procedure was included to minimise the likelihood of hypohydration-related haemoconcentration artificially elevating haemoglobin concentration at the time of assessment. Participants with a urine specific gravity (USG) > 1.021 [36] were reassessed following body composition assessment and, if still above this threshold, following completion of the standardised 600 mL drink; no participant required additional fluid beyond this drink to achieve euhydration (USG ≤ 1.021) prior to haemoglobin sampling.

#### 2.7.2. Physiological and Body Composition Assessments

RMR was measured using a calibrated metabolic cart (TrueOne 2400, Parvo Medics, Salt Lake City, UT, USA). Before each use, the system was calibrated using standard gas concentrations (15.99% O_2_ and 4.00% CO_2_) and a 3 L syringe for volume calibration. Participants rested supine in a thermoneutral (22–24 °C) and dimly lit room for 30 min while wearing a fitted face mask connected to the metabolic cart. During the initial ~20 min, participants rested quietly without gas collection to allow metabolic stabilisation. Expired gases were then collected for 10 min, with the first 5 min discarded and the final 5 min used for analysis. RMR was calculated from oxygen consumption ($\dot{V}$O_2_) and carbon dioxide production ($\dot{V}$CO_2_) using the Weir equation [31]. Steady state was defined a priori as a coefficient of variation for $\dot{V}$O_2_ of <10%, consistent with commonly applied criteria for short-duration RMR assessment protocols in healthy adults [35].

Following the RMR assessment, BM was re-recorded and body composition was assessed using DXA (Hologic Horizon A, Marlborough, MA, USA) following standardised procedures [37]. All scans were performed and analysed by the same licensed bone densitometry operator (FF). Accordingly, DXA-derived total mass may have marginally exceeded scale BM. Although this sequence deviated from pre-scan fasting recommendations of Nana et al. [37], procedures were identical at T1 and T2; therefore, within-participant comparisons were considered acceptable, while absolute body composition values should be interpreted with caution. FFM (lean mass plus bone mineral content) derived from DXA was used as the EA denominator.

#### 2.7.3. Haemoglobin Concentration

Haemoglobin (Hb) concentration was then assessed from a capillary blood sample obtained via fingertip puncture of the ring or middle finger using a sterile single-use lancet. The collection site was cleaned with an alcohol swab, and the first drop was discarded prior to sampling. Samples were collected into microcuvettes and analysed immediately using a portable point-of-care haemoglobin analyser (HemoCue Hb 801 System, HemoCue AB, Ängelholm, Sweden). Sampling was performed only after euhydration had been confirmed to avoid haemoconcentration artefact.

#### 2.7.4. Performance Assessment

Cycling performance was assessed using a standardised CPP test [34] performed on a smart trainer (Tacx Neo 2T, Garmin Ltd., Olathe, KS, USA) to which participants performed the test on their own bicycles to replicate habitual riding position. Following a standardised 5 min warm-up at a perceptually regulated intensity (~100–250 W, RPE 1–4; Borg CR10 scale [38]) including two brief 3 s priming efforts at approximately 70% and 90% of maximal effort, respectively, participants completed six maximal efforts in ascending order of duration: 6 s, 6 s, 15 s, 30 s, 60 s, and 240 s; the 600 s effort of the original protocol and its recovery period were omitted. Active recovery between efforts was 54, 174, 225, 330, and 480 s, respectively, at a light intensity (~100 W). Both 6 s efforts were completed from a standing start, and all subsequent efforts began from a rolling start at 70–80 rpm with self-selected gearing. Participants were allowed to adjust the gear ratio freely during the T0 familiarisation session. The gear ratios self-selected at T0 were recorded and then replicated for each corresponding effort at T1 and T2 to standardise the comparison. Maximal mean power (MMP) was determined for each effort, with MMP over 5 s recorded for the 6 s effort. Critical Power (CP) and the curvature constant (W′) were estimated from the 60 s and 240 s efforts using Golden Cheetah software for macOS (version 3.7, GoldenCheetah.org, Open Source) [39]. Peak $\dot{V}$O_2_ ($\dot{V}$O_2_peak) was determined during the 240 s effort ($\dot{V}$O_2_peak-CPP), with expired air collected continuously through a securely fitted face mask, and was defined as the highest 30 s average oxygen uptake recorded during that effort, obtained from consecutive 30 s bins rather than from a moving average [40]. A maximal effort of this duration exceeds the shortest duration reported to permit attainment of $\dot{V}$O_2_max in trained cyclists [41]. As the protocol was not incremental and neither a verification phase nor plateau criteria were applied, the value is reported as a peak attained during a maximal 240 s effort and not as $\dot{V}$O_2_max. Perceived exertion was recorded immediately after each effort using the Borg CR10 scale [38].

### 2.8. Activity and Well-Being Monitoring

Well-being was monitored each morning throughout, using an adapted version of the Hooper and Mackinnon [42] questionnaire, reformatted as a visual analogue scale (VAS; 0–100) and completed online via the Qualtrics platform. Four items were assessed: sleep quality (0 = very poor, 100 = excellent), muscle soreness, stress, and fatigue (0 = none, 100 = extreme), reflecting perceived recovery and state on waking. The questionnaire also collected bedtime and waking time, from which self-reported sleep duration was derived. A composite Hooper Score was calculated as: (100 − sleep quality) + soreness + stress + fatigue (range 0–400; higher scores indicate poorer well-being).

Session rating of perceived exertion (session RPE) was recorded for each training session using the Borg CR10 scale [38], with participants instructed to record the value upon completion of each session. The highest session RPE recorded on each day was retained for analysis.

Activity energy expenditure (AEE; kcal·day^−1^) was estimated continuously (24 h·day^−1^) using a thigh-worn accelerometer (SENS Motion, SENS Innovation ApS, Copenhagen, Denmark). The accelerometer was affixed to the lateral aspect of the right thigh during the T0 session (Day 1) using anatomical landmarks [43]. The midpoint of the line between the trochanterion and tibiale laterale was identified, then a second point was marked at the midpoint between this point and tibiale laterale (75% of the trochanterion–tibiale laterale distance). The accelerometer’s proximal edge was aligned with this second point and oriented distally toward the knee following the manufacturer’s instructions. The device was worn continuously from this point until removal at the T2 session (Day 23), including during showering and swimming. The device classified each recording epoch into postural and movement categories (sleep, sitting or lying, standing, sporadic walking, walking, moderate activity, running and cycling) and returned the accumulated time spent in each category; it did not provide a direct estimate of energy expenditure. Daily AEE was therefore derived by assigning to each device category a representative MET value drawn from the 2024 Adult Compendium of Physical Activities [44] (Supplementary Table S1), selecting the compendium activity that most closely corresponded to each SENS category, and applying the equation AEE = (MET − 1) × BM × time, where time denotes the accumulated duration in each category expressed in hours and the subtraction of 1 MET isolates the activity-related expenditure above resting metabolism. As these categories describe posture and movement rather than training modality, swimming and resistance exercise were not identified as distinct activities and were allocated by the device algorithm to the categories listed in Supplementary Table S1. AEE was used to characterise 24 h activity and did not contribute to EEE, which was derived from heart rate for all training sessions (Section 2.5). Daily AEE was analysed across complete 24 h periods (Days 2 to 22). Days with fewer than 20 h of valid wear time were excluded from analysis, as were days on which the device returned activity classifications inconsistent with the recorded training (for example, an AEE of zero despite prescribed exercise), which were treated as non-credible. Sleep duration was retained for the full monitoring window (Days 1–23) and used in conjunction with concurrent well-being questionnaire responses. We acknowledge that SENS-derived sleep values should be interpreted cautiously as a device-based estimate of prolonged low-movement or recumbent time rather than polysomnography-derived sleep and may therefore overestimate actual sleep.

### 2.9. Statistical Analysis

All analyses were conducted using Microsoft Excel (version 16.113.4, Microsoft Corporation, Redmond, WA, USA). Given the final sample size (*n* = 3), data were analysed using a within-subject, repeated-measures case-series approach, with emphasis placed on individual responses rather than group-level inference. Daily monitoring data were used to characterise each participant’s intervention exposure. Estimated daily EA was calculated as EI minus EEE, divided by FFM, and expressed as kcal·kg FFM^−1^·day^−1^. Because EI was applied as a constant baseline value, daily EA values are interpreted as estimated rather than directly measured daily exposure. Laboratory and performance outcomes were analysed at the individual level using absolute and relative (% change) differences between pre-intervention (T1) and post-intervention (T2) assessments. These outcomes included RMR, haemoglobin concentration, body composition, maximal mean power outputs, CP, W′, and $\dot{V}$O_2_peak. Baseline testing was used descriptively to provide contextual reference where relevant.

Cross-case comparisons were performed descriptively to identify similarities and differences in achieved EA, adherence to the prescribed exercise stimulus, and physiological and performance responses. Given the exploratory nature of the study and the limited sample size, formal null-hypothesis significance testing was not performed. Instead, emphasis was placed on the magnitude, direction, and consistency of individual responses, as well as temporal patterns observed across the intervention period. Visual inspection of daily trajectories was used to support the interpretation of within-subject variability. This analytical approach was selected to prioritise ecological validity and to capture the complexity and variability inherent to free-living conditions. The primary focus of the analysis was not to establish causal inference, but to characterise real-world variability in the achievement and physiological impact of reduced EA. The selected outcome measures were chosen to capture multiple dimensions of the physiological and performance responses to reduced EA, including RMR, haematological status (haemoglobin), and exercise performance (CPP).

## 3. Results

### 3.1. Participant Characteristics and Study Overview

Three endurance-trained male participants (P1, P2 and P3) completed the study (Table 1); the fourth volunteer withdrew prior to completing the T2 assessments due to unscheduled work commitments unrelated to the intervention. All three remaining participants underwent baseline (T0), pre-intervention (T1), and post-intervention (T2) laboratory assessments, alongside continuous free-living monitoring of training, energy expenditure, and well-being with the 14-day intervention training load and EA exposure presented in Table 2.

**Table 2:** Training load and energy availability exposure during the 14-day intervention.

Participant	EI (kcal)	EI Days 19–22 (kcal·day^−1^)	Required EEE (kcal·day^−1^)	Total EEE (kcal)	Mean Daily EEE (kcal·day^−1^)	Completed EEE (% of Required)	Peak Daily EEE (kcal)	Training Days (*n*/14)	Mean EA (Gross/Net)	SD EA (Gross/Net)	Lowest Daily EA (Gross/Net)	Days EA < 30 (Gross/Net)	Days EA ≤ 20 (Gross/Net)
P1	2200	2178	724	9799	700	97	1775	11	20.3/21.5	7.3/6.5	5.8/9.0	14/14	8/6
P2	2200	2225	949	13,064	933	98	2113	12	20.3/22.2	10.7/9.2	1.4/8.4	10/10	7/6
P3	2900	2863	1750	19,424	1387	79	3374	10	26.3/29.0	20.3/18.0	−8.2/−1.4	7/7	6/5

EI = energy intake; EEE = exercise energy expenditure; EA = energy availability. Gross EA (primary metric) used total session EEE. Net EA subtracted the estimated resting energy expenditure accrued during exercise from gross EEE. Mean daily EEE was calculated across the intervention period. Training days were days with Total EEE > 0 kcal. Threshold counts (<30 and ≤20 kcal·kg FFM^−1^·day^−1^) are descriptive.

### 3.2. EA and Training Load

**Figure 2:**
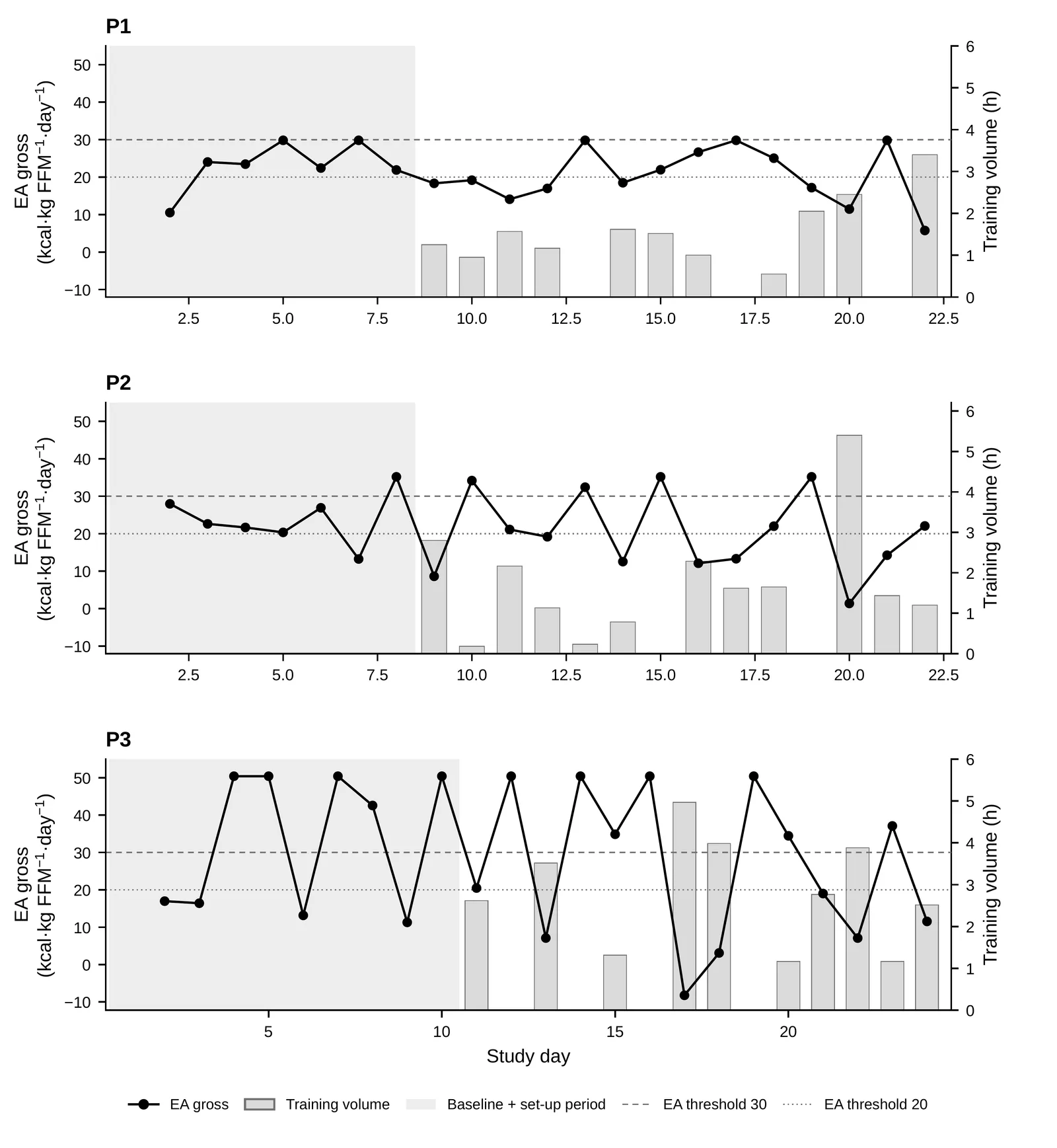
Daily gross energy availability (black line, left y-axis) and training volume (grey bars, right y-axis) for each participant (P1, P2 and P3). Gross EA (EI minus gross EEE, normalised to FFM (kcal·kg FFM^−1^·day^−1^)). The shaded region denotes the baseline and set-up period preceding the 14-day intervention; dashed and dotted lines indicate the 30 and 20 kcal·kg FFM^−1^·day^−1^ thresholds.

EA during the intervention period was not stable despite targeting a consistent reduction in EA; substantial day-to-day variability was observed within each case (Figure 2, Table 2 and Table S2). The within-participant standard deviation of gross EA was 7.3, 10.7 and 20.3 kcal·kg FFM^−1^·day^−1^ for P1, 2 and 3, respectively (net EA: 6.5, 9.2 and 18.0 kcal·kg FFM^−1^·day^−1^).

P1 (Figure 2) showed consistently reduced gross EA (mean 20.3 kcal·kg FFM^−1^·day^−1^), remaining below 30 on all 14 days and at or below 20 on 8 of 14 days. Using net EEE, mean EA was slightly higher (21.5), days at or below 20 decreased to 6, and all 14 days remained below 30.

A mean gross EA of 20.3 kcal·kg FFM^−1^·day^−1^ for P2 (Figure 2) was observed with substantial day-to-day variability (range 1.4 to 35.2), with 10 of 14 days below 30 and 7 of 14 at or below 20. Using net EEE, mean EA was 22.2; days below 30 were unchanged (10 of 14), and days at or below 20 decreased from 7 to 6.

The greatest fluctuation in gross EA (range −8.2 to 50.4 kcal·kg FFM^−1^·day^−1^; mean 26.3) were exhibited by P3 (Figure 2), with 7 of 14 days below 30 and 6 of 14 days at or below 20, indicating alternating periods of substantial deficit and relative energy sufficiency. Net EA was 29.0 (lowest daily value −1.4), with 5 days at or below 20.

These EA profiles were accompanied by distinct training-load patterns (Table 2). Total EEE of 9799 kcal (700 kcal·day^−1^), 13,064 kcal (933) and 19,424 kcal (1387) for P1–3, respectively. Peak daily EEE ranged from 1775 to 3374 kcal across participants, and training days ranged from 10 to 12 of the 14 intervention days. Across the three participants, gross EA was at or below the 20 kcal·kg FFM^−1^·day^−1^ target on 21 of 42 athlete-days (mean 7 of 14 per participant; Figure 2).

**Figure 3:**
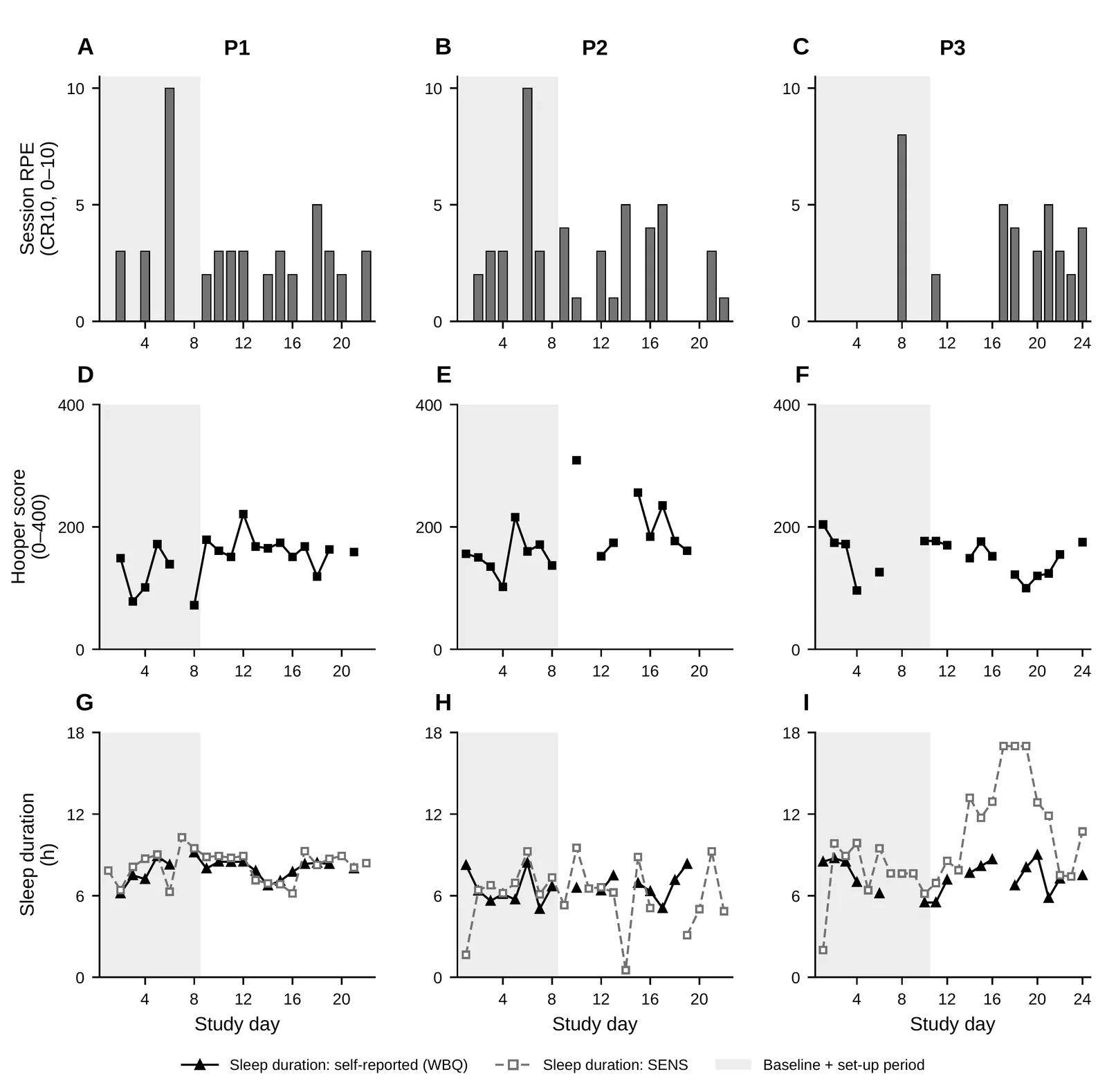
Daily session RPE, Hooper well-being score and sleep duration for each participant. Panels (**A**–**C**): daily session RPE (Borg CR10, 0–10); the isolated peaks on Day 6 (P1, P2) and Day 8 (P3) correspond to the maximal CPP test at T1. Panels (**D**–**F**): composite Hooper Score (0–400) from perceived sleep quality, soreness, stress and fatigue, with higher scores indicating poorer well-being; gaps indicate mornings the questionnaire was not completed. Panels (**G**–**I**): sleep duration from the morning questionnaire (self-reported) and the SENS device. Columns are Participants P1, P2, and P3; the shaded region denotes the baseline and set-up period.

Daily session RPE during the intervention was low to moderate, with mean values of 2.8, 3.0 and 3.5 (range 2 to 5, 1 to 5, and 2 to 5) for P1, 2 and 3, respectively (Figure 3). The isolated peaks before the intervention (RPE 10 on Day 6 for P1 and P2; RPE 8 on Day 8 for P3, whose T1 session took place two days later than scheduled) correspond to the maximal CPP test at T1 and not to a training session.

### 3.3. Physiological Responses

#### 3.3.1. Resting Metabolic Rate (RMR)

Changes in measured RMR (Table 3) from T1 to T2 were small and inconsistent: trivial reductions in P1 (Δ = −0.5%) and P2 (Δ = −0.4%) but marked increase in P3 (1903 to 2204 kcal·day^−1^; Δ = +15.8%).

#### 3.3.2. Haemoglobin and Hydration-Related Measures

Haemoglobin concentration decreased in all three participants between T1 and T2, with reductions ranging from 3.0% to 5.9% (Table 3). Urine specific gravity remained within the pre-specified acceptable range (1.006–1.018) at both time points for all participants, with no individual value exceeding the 1.021 threshold; observed haemoglobin reductions are therefore unlikely to reflect differential hydration status at testing.

#### 3.3.3. Body Composition

Changes in body composition were small and inconsistent across participants, with BM, FFM or fat mass (FM) all changing by ≤0.8 kg (Table 3).

### 3.4. Performance Responses

#### 3.4.1. Maximal Mean Power and CP

Performance changes from T1 to T2 were heterogeneous, with no consistent directional pattern (Table 3). P1 showed small reductions in short-duration power (MMP5s–MMP60s), but improved MMP240s and CP, while W′ decreased. P2 recorded substantial reductions in high-intensity performance (MMP5s, MMP15s), mixed longer-duration responses and a decrease in CP, with W′ unchanged. P3 showed small short-duration changes but improved MMP240s and CP, with no change in W′.

#### 3.4.2. $\dot{V}$O_2_peak and Perceived Exertion

Peak $\dot{V}$O_2_ ($\dot{V}$O_2_peak-CPP) was essentially unchanged in P1 (+0.2%) and increased in P2 (+12.1%) and P3 (+4.3%) from T1 to T2 (Table 3). Ratings of perceived exertion (Borg CR10) ranged from 2 to 10 across the six maximal efforts and remained stable between time points, differing by no more than two units for any matched effort within each participant.

### 3.5. Daily Monitoring Responses

#### 3.5.1. Sleep and Well-Being

P1 recorded a moderate increase in mean Hooper Score from baseline (125 ± 34 arbitrary units (a.u.)) to intervention (165 ± 24 a.u.), while sleep duration remained relatively stable across both questionnaire- and SENS-derived estimates (~8.0 and 8.1 h·night^−1^, respectively).

Well-being in P2 decreased during the intervention, with mean Hooper Score increasing from baseline (151 ± 44 a.u.) to intervention (206 ± 52 a.u.). Two days showed markedly poorer well-being were observed (Hooper Score = 309 and 256 on Days 2 and 7, respectively), coinciding with periods of higher recent training load. This participant also showed the greatest variability in self-reported sleep duration (mean 7.0 h·night^−1^, several nights below 6 h), whereas SENS-derived estimates were lower and more variable (mean 5.5 h·night^−1^).

P3 observed no meaningful change in composite well-being (baseline: 147 ± 39 a.u.; intervention: 147 ± 27 a.u.). Self-reported sleep averaged 7.4 h·night^−1^, whereas SENS-derived estimates were substantially higher (11.3 h·night^−1^), suggesting that the device may have captured prolonged recumbent or low-movement periods rather than sleep alone.

Well-being questionnaires were completed on 12 of 14 intervention days for P1 (missing Days 12 and 14), 8 of 14 days for P2 (missing Days 1, 3, 6, 12, 13 and 14), and 11 of 14 days for P3 (missing Days 3, 7 and 13). Participants attributed the missed questionnaires to forgetting. Hooper Score and sleep duration across the monitoring period are shown for each participant in Figure 3. Agreement between questionnaire- and device-derived sleep duration was poor and varied between participants (Supplementary Figures S1 and S2).

#### 3.5.2. Free-Living Activity Energy Expenditure

Device-based activity energy expenditure (AEE), estimated from the time spent in each SENS activity category scaled by BM, varied substantially within and between participants. Valid wear covered all 14 days for P1 (mean AEE 1325 kcal·day^−1^; range 610 to 2395), but only 6 of 14 days for P2 (mean on valid days 1464 kcal·day^−1^; range 886 to 2023). P2 attributed the non-wear days to detachment of the sensor; on those days the device recorded fewer than 20 h and they were classified as non-valid (Section 2.8). For P3, wear time met the validity threshold on all 14 days; however, on three consecutive days (Days 7 to 9) the device classified an implausibly ~17 h·day^−1^ as recumbent or sleep and returned zero AEE, despite high prescribed cycling loads on Days 7 and 8 (gross EEE of 3374 and 2722 kcal, respectively) and no structured exercise on Day 9. These non-credible days were excluded, leaving 11 valid days (mean AEE 980 kcal·day^−1^; range 174 to 1977). Across participants, daily AEE tended to increase with concurrent gross EEE, indicating that the thigh-worn accelerometer captured locomotor exercise within its activity categories. Accordingly, SENS-derived AEE represents total ambulatory activity overlapping structured EEE, rather than non-exercise activity, and provided no clear evidence of movement compensation on high-training days.

## 4. Discussion

### 4.1. Principal Findings

The primary aim was to characterise the extent to which a prescribed reduction in EA could be achieved and sustained under free-living conditions, and to describe the within- and between-participant variability in achieved EA across a 14-day intervention targeting 20 kcal·kg FFM^−1^·day^−1^ in male endurance athletes. A secondary aim was to describe the associated physiological and performance responses. Reductions in estimated EA were achieved across all participants, but the magnitude and consistency varied substantially; the intervention did not produce a uniform LEA stimulus, but participant-specific exposure profiles differed in magnitude, variability, and training load. This illustrates why a case-series design, rather than a group-averaged trial, was necessary: exposure to a nominally standardised intervention was not itself standardised across participants, a pattern a between-group mean would obscure [45]. Physiological responses were similarly heterogeneous. RMR displayed no consistent direction, haemoglobin decreased only modestly in all participants and body composition changes were minimal and within the technical error of DXA (~0.5–1.0 kg for FFM and fat mass in athletes) so should not be interpreted as meaningful. This absence of a uniform group-level signal is consistent with a design constructed to describe individual-level response patterns that group-based designs cannot resolve [26]. However, due to the case-series design, the absence of signal change cannot be separated from the small sample effect, and the findings are best regarded as hypothesis-generating, consistent with case-study reporting standards in sport science [27].

At the individual level, P1 exhibited the most consistent pattern of reduced EA with minimal physiological change and mixed performance outcomes. Whilst P2 observed greater signs of physiological and functional strain, including the largest reduction in haemoglobin concentration, reduced well-being, greater sleep variability, and reduced performance capacity across several power outputs. In comparison, P3 presented with the greatest intra-individual variability in EA, characterised by alternating periods of pronounced deficit and relative sufficiency, yet concurrently maintained or improved multiple performance indices despite sustaining the highest cumulative training load. Recalculation using net exercise energy expenditure increased absolute EA values modestly but did not materially alter this overall interpretation. Collectively, these results highlight that both the magnitude and temporal profile of EA exposure, as well as the associated physiological and performance responses, exhibited substantial inter-individual variability under free-living conditions. Thereby underscoring the considerable challenges associated with experimentally inducing and detecting LEA in such an ecologically valid context.

### 4.2. Achieving Sustained LEA in Free-Living Conditions

A key observation was the difficulty of achieving sustained EA reductions consistently across participants (Figure 2). All individuals experienced periods of low estimated EA, reaching the 20 kcal·kg FFM^−1^·day^−1^ target on a mean of 7 of 14 days, but these were frequently interspersed with higher EA days (Figure 2), reflecting day-to-day variability in EEE and free-living behaviours. This aligns with previous work highlighting the methodological difficulty of quantifying and interpreting EA under free-living conditions, where variation in exercise load, spontaneous activity, and dietary intake can substantially influence estimated exposure [15,16,20]. Free-living EA in male endurance athletes varies widely; Taylor et al. [29] observed a 7-day mean of 19.5 ± 9.1 kcal·kg FFM^−1^·day^−1^ (daily range −21.9 to 76.0) in elite road cyclists, whereas Vardardottir et al. [46] reported a weekly mean of 33.4 ± 11.8 (range 11.6 to 57.0) in male athletes, with no athlete below the LEA threshold every day. Our cases are consistent with this variability (mean: 20.3 to 26.3; range: −8.2 to 50.4). As habitual EI was held constant by design, all variation in estimated EA arose from variation in completed EEE, whether from adherence to the prescribed programme, day-to-day structure of the prescription itself or error in HR-derived EEE, and these sources cannot be separated with the present data. Day-to-day variation in actual intake, which was not measured during the intervention, and which may increase on high-training load days, would add to the variability in true EA rather than reduce it. These findings suggest that studies assuming stable LEA from prescribed protocols may overestimate the consistency of energy deficiency under real-world conditions, particularly when the number of days on which reduced EA is actually achieved is not reported, with important implications for research design and the ecological validity of threshold-based approaches.

The distribution of EA differed markedly between participants. A highly bimodal profile was exhibited by P3, with rest days approaching 50 kcal·kg FFM^−1^·day^−1^ and high-volume days as low as −8.24 kcal·kg FFM^−1^·day^−1^ (Day 7). On that day, gross EEE (3374 kcal) exceeded the constant EI applied in the calculation (2900 kcal) by 474 kcal, giving EA = (2900 − 3374)/57.51 = −8.24 kcal·kg FFM^−1^·day^−1^. A negative EA indicates only that estimated exercise expenditure exceeded intake. It is not an estimate of whole-body energy balance, which would additionally require RMR, non-exercise activity and diet-induced thermogenesis, none of which enter the EA calculation. Given that errors in estimating EI and EEE in free-living athletes can each be 300–600 kcal·day^−1^ [15], such an extreme value may partly reflect the compounded uncertainty of estimating both EI and EEE under free-living conditions rather than a physiologically sustained deficit of that magnitude. Although mean EA (26.3, gross) did not consistently fall below the study design target of 20, the large intra-individual variability raises a key question: are LEA’s physiological effects determined more by cumulative mean exposure or by the acute severity and frequency of discrete low-EA days? This distinction remains insufficiently explored in laboratory-based LEA models and represents an important gap in understanding how free-living EA patterns translate into physiological outcomes.

A further consideration is the use of gross EEE for the primary EA calculation. While this approach reflects the total energetic cost of exercise and matches the outputs of commercial athlete-monitoring platforms, it likely overestimates the exercise energy relevant to the original EA construct, which net EEE more closely approximated. As a sensitivity analysis, we also computed net EA; this yielded higher absolute estimates and fewer days below conventional thresholds but did not materially alter the pronounced intra- and inter-individual variability. Because habitual EI was assumed constant across days, all day-to-day variability in estimated EA was, by construction, attributable to variation in completed EEE. As true daily EI also fluctuates under free-living conditions, this approach may underrepresent the total variability in actual EA exposure. Interpretation of free-living EA therefore depends not only on the magnitude of intake and expenditure, but also on how EEE is operationalised and how exposure is distributed over time.

### 4.3. Resting Metabolic Rate Responses

Measured RMR did not exhibit a consistent directional response across participants. Whereas P1 and P2 recorded only trivial reductions following the intervention, P3 demonstrated an increase in RMR. Notably, P3 accumulated a substantial training load over the final four intervention days (total EEE: ~7300 kcal across Days 11–14), including a high-volume session on the day before post-intervention testing. However, the magnitude of the observed increase (+15.8%) exceeds the typical day-to-day biological and technical variability of RMR measurement (~5–10% CV) [35] and may therefore reflect residual physiological perturbation from the preceding training load, incomplete adherence to pre-test conditions, or a combination of both. Accordingly, this observation should be interpreted with caution and cannot be attributed to a change in basal metabolic rate. As no exercise-free day preceded T2, the elevation may reflect residual effects of the preceding session (elevated post-exercise metabolism, incomplete recovery, altered hydration or glycogen status, or muscle damage), and it should not be interpreted as a sustained metabolic adaptation to LEA. Collectively, our findings do not support a uniform pattern of RMR suppression in response to the present free-living LEA intervention.

The lack of consistency is unsurprising given the methodological and physiological complexity of studying LEA under free-living conditions. Daily variation in EI, EEE, and recovery behaviours may mask clear metabolic responses, especially over short interventions. This aligns with previous work highlighting the difficulty of quantifying and interpreting EA in free-living athletes [15,20] and with broader evidence that metabolic responses to LEA vary across prospective studies or athlete populations [22].

Importantly, interpretation in the present study was based on directly measured RMR derived from indirect calorimetry rather than on predicted-to-measured RMR ratios. This distinction is relevant because the accuracy of prediction equations in athletic populations is variable, and caution has been advised when using such ratios to infer LEA-related metabolic suppression [47]. Our position is also aligned with the current IOC REDs framework, which does not support reliance on any single marker as a definitive indicator of LEA or REDs [14].

### 4.4. Performance Responses: Evidence of Heterogeneity

Performance outcomes varied markedly between participants, with no consistent direction of change. P2 recorded modest reductions in multiple performance metrics, including a decrease in CP from 229 to 219 W (T1 vs. T2; −4.4%), whereas P3 maintained or improved performance despite repeated periods of very low EA within a highly variable exposure profile. These findings are consistent with previous research indicating the relationship between LEA and exercise performance is not uniform and may depend on the severity, duration, and context of the energy deficit, as well as the specific performance outcome assessed [12,13,22].

Differences in performance responses may not only relate to training-load but also to broader contextual factors. Age varied substantially between the three participants (18.8 to 49.2 years), but the case-series design precludes meaningful interpretation of age effects. The participant who maintained or improved several performance outcomes accumulated the highest total and mean daily EEE, whereas the participant whose performance declined did so under a lower overall load. Given the design and heterogeneous exposure, we interpret this descriptively rather than causally. Divergent changes within a participant, such as the increase in $\dot{V}$O_2_peak alongside reductions in MMP and CP in P2, do not indicate uniform improvement or impairment and cannot be attributed specifically to reduced EA. Over 14 days without a time-matched control condition, they may reflect any combination of training stimulus, accumulated fatigue, recovery, familiarisation, body-mass change, pacing and measurement variability. Training structure also differed: the highest-performing participant combined high-magnitude sessions with distinct recovery periods, whereas others used more evenly distributed, lower-intensity training. Whether well-being contributed cannot be determined, although the descriptive pattern is consistent (Figure 3). The participant whose performance declined (P2) reported the worst well-being (mean Hooper Score 206 a.u.) and an elevated score on the morning of post-intervention testing, whereas the participant who maintained or improved performance (P3) reported the most stable well-being (mean 147 a.u.). This interpretation is constrained by missing well-being data in the days immediately preceding post-intervention testing, particularly for P2, who provided no responses for the final intervention days.

P3 also differed in age and competitive calibre from the other cases, being the youngest and only Tier 3 [28] athlete. Although the present design does not permit inference regarding the contribution of these characteristics, they represent important contextual factors when interpreting his apparent tolerance of high training load and performance maintenance despite marked variability in EA exposure. These characteristics warrant greater consideration in future experimental designs, as the interplay between rest, recovery, refuelling, sleep, and training load, particularly in higher-calibre athletes, is where practical insight into EA exposure, adaptation to LEA, and performance outcomes is most needed.

These findings suggest that both the magnitude and distribution of training load may influence the extent to which reductions in EA impact performance. Within this framework, EA may act as a modulating factor, with its effects depending on the interaction with training stimulus, recovery dynamics, and athlete calibre with each potentially representing inter and intra individual inflection points or thresholds of action. While speculative, endothelial cells may represent an emerging mediator of athlete responses to LEA, given their role in sensing exercise-induced mechanical and metabolic stress, coordinating intra- and inter-organ communication, and supporting metabolic homeostasis [48]. The maintenance or improvement of performance despite reduced EA may therefore reflect a dissociation between short-term functional capacity and underlying physiological strain. Conversely, the observed performance decline in one participant suggests that when reductions in EA coincide with a less favourable balance between training load and recovery, performance impairments may become more evident.

### 4.5. Haemoglobin Reductions and Physiological Strain

One of the few consistent findings between participants was a modest reduction in haemoglobin concentration. Although the magnitude of change was small, this may reflect an early physiological response to the intervention. Importantly, hydration status was screened immediately prior to sampling, and all urine specific gravity values were within the pre-specified acceptable range at both testing time points. Accordingly, the observed reductions in haemoglobin are unlikely to be attributable to hypohydration-related haemoconcentration at the time of testing. Within the broader REDs framework, such findings should be interpreted cautiously, as no single marker provides a definitive indication of LEA-related physiological disruption and responses may vary across individuals and systems [14].

### 4.6. Well-Being and Behavioural Responses

Visual inspection of the daily EA trajectories (Figure 2) and the well-being and sleep data (Figure 3) suggested that the factors associated with well-being differed between cases. In P1 and P2, poorer well-being tended to follow days characterised by higher exercise energy expenditure and lower estimated EA, whereas in P3, well-being appeared more closely aligned with sleep duration. Although these patterns should be interpreted cautiously given the small number of observations and missing questionnaire data, they support the view that responses to reduced EA in free-living settings are shaped by individual context rather than by a single dominant factor, with competition level and the associated training volume likely also influencing the size of the energy deficit and the athlete’s capacity to recover [29,49].

### 4.7. Inter-Individual Variability and the Interpretation of EA

A key finding of this study is the substantial inter-individual variability across all outcomes. Participants differed in the magnitude and consistency of EA reduction, and in physiological, performance, and behavioural responses, with no consistent pattern except a modest reduction in haemoglobin concentration. This variability challenges the assumption that a standard intervention produces comparable exposure and responses in all individuals, and supports growing recognition that individual context, such as behavioural patterns, training characteristics, and physiological resilience, critically shapes the response to reduced EA [14].

Critically, mean EA did not adequately characterise individual exposure. Although P1 and P2 had similar average EA, their daily profiles differed markedly in variability and timing and were linked to distinct physiological and performance responses. This has key implications for research design and practice, where relying on mean EA may obscure meaningful day-to-day fluctuations, particularly when training load and recovery behaviours vary concurrently. EA should therefore be interpreted not just as a single summary value, but as a dynamic, time-varying exposure shaped by EI, EEE, and behavioural context. Detecting and monitoring LEA may be improved by methods that consider within-subject variability and temporal patterns, rather than relying solely on threshold classifications from mean EA. For practitioners, we propose three implications to practice suggested by our interpretation. Firstly, appreciate that a 14-day mean EA can conceal single days of severe deficit, so the distribution of daily values should be examined alongside the mean. Secondly, acknowledge that a prescribed EA target should not be assumed to have been reached without daily monitoring of EEE, because the same prescription produced widely different exposures in the three athletes reported here. Third and final, EA estimated with a constant intake value is a monitoring index rather than a diagnostic measure and should not be used on its own for decisions concerning athlete health.

### 4.8. Strengths and Limitations

A key strength is the integration of laboratory-based assessments with continuous free-living monitoring of EEE and physical activity across physiological, performance, and well-being domains, providing an ecologically valid framework. Completed EEE was compared daily with the individual requirement against a pre-specified criterion, laboratory procedures were identical at T1 and T2, including control of hydration before haemoglobin sampling, gross and net EA are both reported as a sensitivity analysis, and all three cases are reported in full, including the days on which the prescription was not met and the days with missing data. Several limitations apply. Firstly, regarding study design decisions, the reported sample size (*n* = 3) limits generalisability and precludes statistical inference, consistent with the case-series design. Recruitment and retention are particularly challenging given the high participant burden and prolonged exposure required. The approved protocol targeted *n* = 15, derived from an a priori estimate of *n* = 12 for a repeated-measures design, allowing for 20% attrition. While four participants were enrolled, one withdrew and the three cases differed in age (18.8 to 49.2 years) and competitive calibre (Tier 2 and Tier 3). Regarding the study design, no time-matched control condition was included, although athletes maintained their habitual training and diet during the baseline and set-up period preceding the intervention (Figure 1), so that the T1 assessment served as a within-participant control. We also acknowledge that, in an exercise-induced LEA model, the energy deficit cannot be separated from the training load that produced it, so the findings are hypothesis-generating and permit no inference about the effects of LEA. Further, EI was held constant at the habitual value by design and was not measured on 10 of the 14 intervention days, so a compensatory increase in intake on high-load days cannot be excluded. By design, EEE was estimated using proprietary device algorithms (Garmin/TrainingPeaks) reporting gross energy expenditure, which includes the resting component accrued during the session and thus likely overestimates EEE and underestimates EA. Although the adjustment rule was pre-specified, the days on which the prescription was adjusted were not recorded. Achieved daily EA is consequently an estimate whose error compounds the errors in EI and EEE [15], and single-day values should be read with that uncertainty in mind. We also recognise that the DXA scans were not performed fasted (a standardised 600 mL carbohydrate-electrolyte drink was consumed before and after), deviating from Nana et al. [37]; within-participant comparisons remain valid given identical T1 and T2 procedures, but absolute values should be interpreted accordingly. We also note that valid SENS data were available on only 6 of 14 days for P2, and for P3 the device returned implausible activity classifications on three consecutive days (~17 h·day^−1^ recumbent or sleep, AEE of zero), despite high prescribed cycling on two of them; these days were therefore excluded, indicating that wear time alone was insufficient to confirm validity and that the thigh-worn accelerometer did not reliably capture cycling. Consequently, no statement about non-exercise activity during the intervention can be made for P2, and because his well-being questionnaires were completed on 8 of 14 days with none in the final three days, his well-being immediately before T2 is unknown. Furthermore, the SENS-derived AEE was not measured directly but estimated by assigning a single MET value from the 2024 Adult Compendium of Physical Activities [44] to each activity. Because the device classifies activity type but not intensity, a fixed MET cannot capture within-category intensity, most consequentially for running and cycling, likely underestimating the energy cost of high-intensity endurance training. SENS-derived AEE should therefore be interpreted as an approximate index of physical activity rather than a precise estimate of energy expenditure.

Although a familiarisation session (T0) preceded pre-intervention testing, a single exposure may not have stabilised pacing during the CPP, particularly the 240 s effort from which CP and W′ were derived. In at least one participant, the change in CP from T0 to T1 was comparable to that from T1 to T2, suggesting familiarisation effects may not have fully plateaued. Changes in CP and W′ from T1 to T2 should therefore be interpreted cautiously, as residual learning effects cannot be excluded. CP and W′ were also estimated from two efforts rather than the three of the source protocol [34], which leaves the linear model exactly determined, and changes in power output should be read against the typical error of the test, 3.6 ± 0.8% across effort durations [34], within which several of the individual changes fall. $\dot{V}$O_2_peak-CPP was derived from consecutive 30 s bins rather than from a moving average, and processing strategies of this kind can differ by up to 7% at the individual level [40]; as the respiratory exchange ratio did not exceed 1.00 in any assessment, these values represent the highest oxygen uptake attained during a maximal 240 s effort rather than maximal aerobic capacity. Training load was also unevenly distributed: P3 accumulated a disproportionate share of total EEE in the final four days, which may have influenced T2 outcomes independently of cumulative EA exposure. No exercise-free day preceded T2, so post-intervention RMR and performance may carry residual effects of the final training days. Finally, the 14-day duration may have been insufficient to elicit consistent physiological responses, as some LEA-related markers may require more prolonged exposure than was achieved.

Several of these limitations have direct solutions. Future studies of this design should include a run-in of at least 7 days with measured EI and EEE; record EI on more intervention days, using low-burden image-based methods to limit the decline in compliance; derive EEE from power-meter data with individual calibration and subtraction of the resting component; pre-register the tolerance band and the adjustment rule and log every adjustment; impose a standardised exercise-free day before each laboratory session; provide two familiarisation sessions for the CPP; record the reasons for missing data at each contact; and report the number of days on which the target was reached as the primary feasibility outcome.

## 5. Conclusions

In this pilot case series, a prescribed reduction in EA to 20 kcal·kg FFM^−1^·day^−1^ was reached on a mean of 7 of 14 intervention days, and daily exposure varied widely both within and between athletes. Physiological and performance responses were correspondingly inconsistent, and with three participants, no conclusion can be drawn about the effects of LEA itself. Inducing and verifying a target EA under free-living conditions is therefore feasible only in the limited sense that the mean can be moved. The exposure that results is not uniform, and the mean EA alone does not describe it. Future studies should pre-register the tolerance band and the load-adjustment rule, measure EI on every intervention day rather than in sampled windows, standardise run-in and pre-test recovery periods, and report the number of days on which the prescribed target was achieved as a primary feasibility outcome.

## Figures and Tables

**Table 1:** Participant characteristics.

Participant	Age (y)	Primary Sport	Athlete Classification	BM (kg)	FFM (kg)	FM (kg)
P1	49.2	Triathlon	Tier 2 (Trained/Developmental)	90.8	73.8	18.8
P2	22.6	Triathlon	Tier 2 (Trained/Developmental)	72.8	62.5	11.0
P3	18.8	Cycling	Tier 3 (Highly Trained/National Level)	64.9	57.5	9.0

BM = body mass; FFM = fat-free mass; FM = fat mass. Athlete classification is based on the Participant Classification Framework [28]. BM, FFM and FM were measured at T1.

**Table 3:** Changes in physiological and performance outcomes from pre- (T1) to post-intervention (T2).

Variable	P1	P2	P3
RMR (kcal·day^−1^)	1683 → 1675 (−0.5%)	1935 → 1927 (−0.4%)	1903 → 2204 (+15.8%)
Haemoglobin (g·L^−1^)	153 → 148 (−3.3%)	153 → 144 (−5.9%)	168 → 163 (−3.0%)
BM (kg)	90.80 → 90.00 (−0.80)	72.75 → 72.20 (−0.55)	64.85 → 65.35 (+0.50)
FFM (kg)	73.79 → 73.16 (−0.63)	62.55 → 62.83 (+0.28)	57.51 → 57.70 (+0.19)
Fat mass (kg)	18.77 → 19.21 (+0.44)	11.01 → 10.48 (−0.53)	9.04 → 9.19 (+0.15)
$\dot{V}$O_2_peak-CPP (mL·kg^−1^·min^−1^)	44.12 → 44.19 (+0.2%)	58.99 → 66.12 (+12.1%)	81.91 → 85.40 (+4.3%)
MMP 5 s (W)	1092 → 1083 (−0.8%)	870 → 730 (−16.1%)	936 → 921 (−1.6%)
MMP 15 s (W)	631 → 586 (−7.1%)	737 → 660 (−10.5%)	842 → 847 (+0.6%)
MMP 30 s (W)	530 → 512 (−3.4%)	545 → 568 (+4.2%)	617 → 607 (−1.6%)
MMP 60 s (W)	391 → 381 (−2.6%)	416 → 404 (−2.9%)	508 → 515 (+1.4%)
MMP 240 s (W)	241 → 252 (+4.6%)	276 → 265 (−4.0%)	355 → 371 (+4.5%)
Critical Power (W)	191 → 209 (+9.4%)	229 → 219 (−4.4%)	304 → 323 (+6.3%)
W′ (kJ)	12 → 10 (−16.7%)	11 → 11 (0.0%)	12 → 12 (0.0%)

BM = body mass; FFM = fat-free mass; CPP = cycling power profile; MMP = maximal mean power; W′ = work above critical power. Values are T1 → T2, with the percentage change in parentheses, except body composition, for which the absolute change (kg) is shown. $\dot{V}$O_2_peak-CPP is the highest 30 s average oxygen uptake during the 240 s CPP effort.

## Data Availability

The data presented in this study are available upon request from the corresponding author due to privacy restrictions.

## Supplementary Materials

The following supporting information can be downloaded at https://www.mdpi.com/article/10.3390/sports14100446/s1, Figure S1: Within-participant associations of device-measured (SENS) sleep duration with self-reported sleep duration (panels A to C) and self-reported sleep quality (panels D to F); Figure S2: Bland–Altman comparison of self-reported (questionnaire) and device-measured (SENS) sleep duration, pooled across participants (panel A) and by participant (panels B to D); Table S1: Correspondence between SENS Motion activity categories and 2024 Adult Compendium of Physical Activities metabolic equivalent (MET) values used to estimate activity energy expenditure (AEE); Table S2: Daily energy intake (EI) used and recorded, fat-free mass (FFM), required, prescribed and completed exercise energy expenditure (EEE) and achieved energy availability (EA) for each participant across the 14-day intervention.

## Abbreviations

The following abbreviations are used in this manuscript:

LEA	low energy availability
EA	energy availability
EI	energy intake
EEE	exercise energy expenditure
AEE	activity energy expenditure
FFM	fat-free mass
FM	fat mass
BM	body mass
RMR	resting metabolic rate
DXA	dual-energy X-ray absorptiometry
Hb	haemoglobin
USG	urine specific gravity
HR	heart rate
mFR	mobile food record
MET	metabolic equivalent of task
RPE	rating of perceived exertion
VAS	visual analogue scale
CP	critical power
W′	work above critical power
MMP	maximal mean power
CPP	cycling power profile
$\dot{V}$O_2_peak	peak oxygen uptake
REDs	Relative Energy Deficiency in Sport
IOC	International Olympic Committee
ISAK	International Society for the Advancement of Kinanthropometry
SENS	SENS Motion (thigh-worn accelerometer)
T0/T1/T2	familiarisation/pre-intervention/post-intervention testing
