# Supplementary material for: Variable Energy Availability Exposure and Heterogeneous Physiological Responses to a Prescribed Low Energy Availability Intervention in Free-Living Male Endurance Athletes: A Pilot Case-Series
Source: Sports (Basel). 2026 Oct 9;14(10):446. doi: 10.3390/sports14100446 (PMC13709522; doi:10.3390/sports14100446)
Supplement: Supplementary file 1 [file sports-14-00446-s001.zip › sports-4496680-supplementary.pdf]

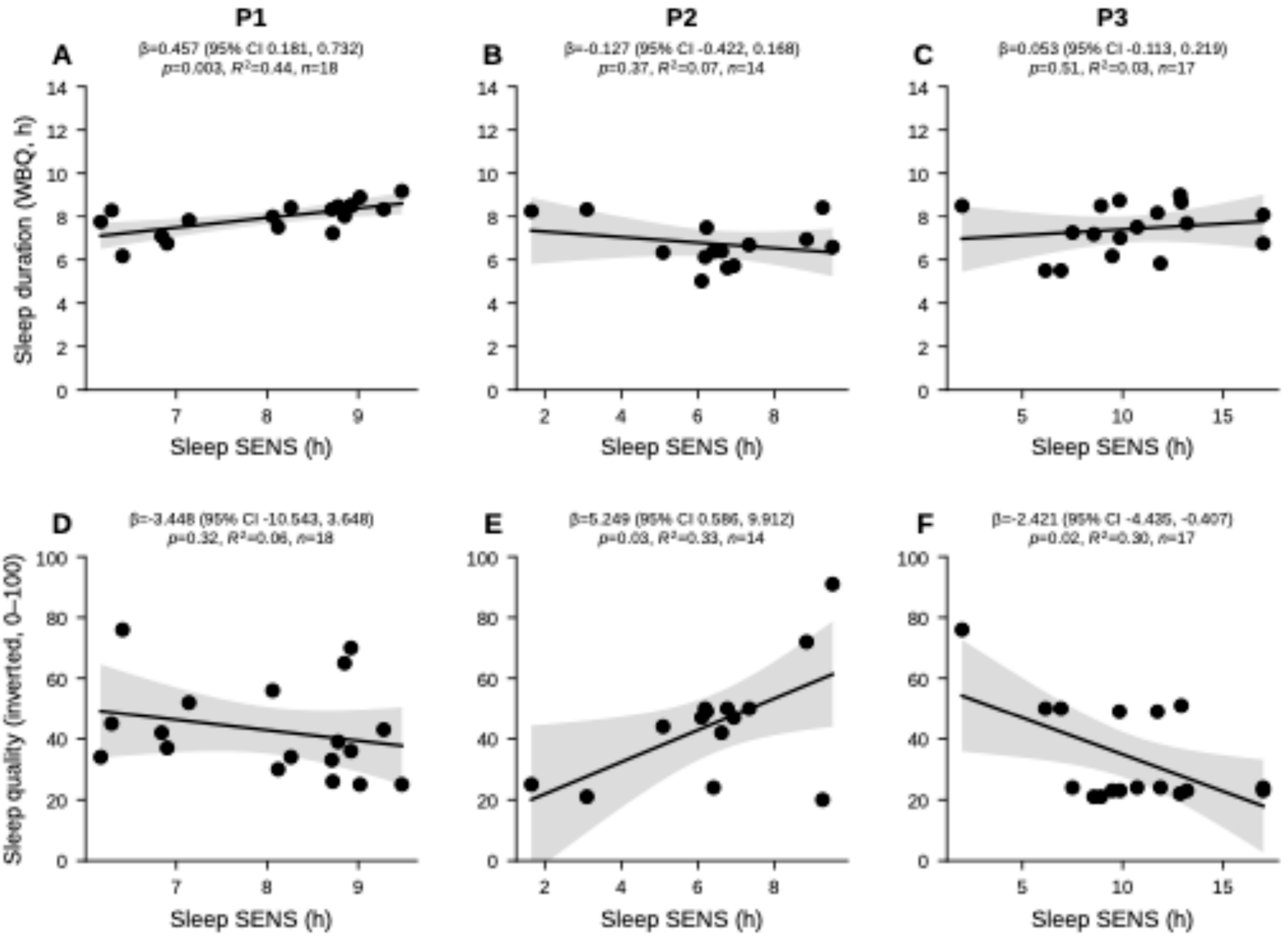

Supplementary Figure S1. Within-participant associations of device-measured (SENS) sleep duration with self-reported sleep duration (panels A to C) and self-reported sleep quality (panels D to F). Each panel shows the linear association for one participant, with the regression coefficient, 95% confidence interval, *p*-value, coefficient of determination, and number of paired observations. Self-reported sleep quality is expressed on an inverted 0–100 scale, with higher values indicating poorer quality. Associations are presented descriptively and do not constitute a measure of agreement.

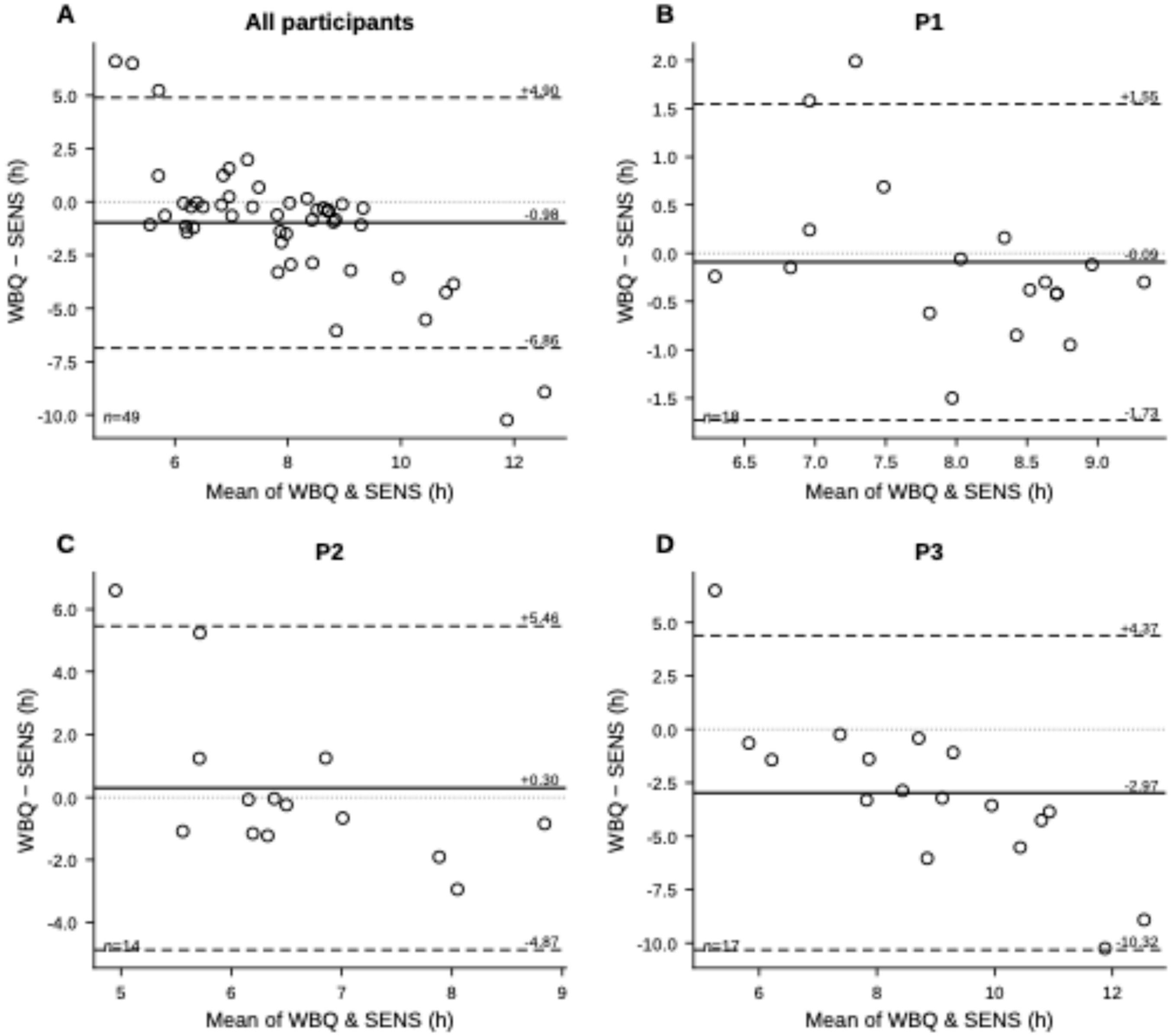

Supplementary Figure S2. Bland-Altman comparison of self-reported (questionnaire) and device-measured (SENS) sleep duration, pooled across participants (panel A) and by participant (panels B to D).

The solid line indicates the mean difference and the dashed lines the 95% limits of agreement. The pooled analysis (panel A) does not account for repeated measures within participants and is presented descriptively; the participant-specific limits of agreement (panels B-D) are the primary comparison.

Supplementary Table S1. Correspondence between SENS Motion activity categories and 2024 Adult Compendium of Physical Activities metabolic equivalent (MET) values used to estimate activity energy expenditure (AEE).

SENS activity category	Compendium code	MET	Net MET (MET − 1)	Corresponding compendium activity
sleep_no_movement	07030	1.0	0.0	Sleeping
sleep_movement	07030	1.0	0.0	Sleeping
sleep_active	07011	1.0	0.0	Lying quietly, awake
lying_sitting_rest	07021	1.0	0.0	Sitting quietly, general
lying_sitting_movement	07022	1.5	0.5	Sitting, fidgeting
upright_stand	07040	1.3	0.3	Standing quietly
upright_sporadic_walk	11600	1.8	0.8	Standing tasks, light effort
upright_walk	11792	3.8	2.8	Walking on the job, 2.8 to 3.4 mph, moderate speed
upright_moderate	17255	4.0	3.0	Walking, self-selected speed, firm surface
upright_run	12029	8.5	7.5	Running, 5.0 to 5.2 mph (12 $min \cdot mile^{-1}$)
cycling	01040	10.0	9.0	Bicycling, 14 to 15.9 mph, fast, vigorous effort

AEE = activity energy expenditure; MET = metabolic equivalent of task; SENS = SENS Motion thigh-worn accelerometer (SENS Innovation ApS, Denmark). MET values were assigned to each device-classified activity category from the 2024 Adult Compendium of Physical Activities [44], selecting the compendium activity that most closely corresponded to each category. Daily AEE was estimated as the sum across categories of (MET − 1) × BM (kg) × time (h), where the subtraction of 1 MET isolates the energy expended above resting metabolism (1 MET ≈ 1 $kcal \cdot kg^{-1} \cdot h^{-1}$). A single representative MET value was applied per category; because the device classifies activity type but not exercise intensity, these values represent approximations and may not reflect within-category intensity variation, most notably for the running and cycling categories in well-trained endurance athletes. The categories are

those returned by the device and do not correspond to training modalities; swimming and resistance exercise have no category and were allocated by the device algorithm to the categories listed, so AEE on those days is likely to be underestimated. EEE for all training modalities was derived separately from heart rate (Section 2.5).

Supplementary Table S2. Daily energy intake (EI) used and recorded, fat-free mass (FFM), required, prescribed and completed exercise energy expenditure (EEE) and achieved energy availability (EA) for each participant across the 14-day intervention.

Day	Date	Recorded EI (kcal)	Prescribed cycling EEE (kcal)	Completed cycling EEE (kcal)	Completed other-modality EEE (kcal)	Total completed EEE (kcal)	Achieved gross EA (kcal·kg FFM^{-1}·day^{-1})
**P1: EI used 2200 kcal·day^{-1}; FFM 73.8 kg; required EEE 724 kcal·day^{-1}**							
1	18 Nov	–	847	847	0	847	18.3
2	19 Nov	–	0	0	785	785	19.2
3	20 Nov	–	817	1160	0	1160	14.1
4	21 Nov	–	475	0	947	947	17.0
5	22 Nov	–	995	0	0	0	29.8
6	23 Nov	–	995	835	0	835	18.5
7	24 Nov	–	734	579	0	579	22.0
8	25 Nov	–	0	0	233	233	26.7
9	26 Nov	–	817	0	0	0	29.8
10	27 Nov	–	917	0	352	352	25.0
11	28 Nov	1900	817	932	0	932	17.2
12	29 Nov	2416	1445	1354	0	1354	11.5
13	30 Nov	2056	734	0	0	0	29.8
14	01 Dec	2341	1602	1006	769	1775	5.8
**Mean**		**2178**	**800**	**480**	**220**	**700**	**20.3**
**P2: EI used 2200 kcal·day^{-1}; FFM 62.5 kg; required EEE 949 kcal·day^{-1}**							
1	25 Nov	–	900	840	820	1660	8.6
2	26 Nov	–	0	0	63	63	34.2
3	27 Nov	–	900	740	139	879	21.1
4	28 Nov	–	0	0	1000	1000	19.2
5	29 Nov	–	1773	0	173	173	32.4
6	30 Nov	–	0	709	706	1415	12.6
7	01 Dec	–	0	0	0	0	35.2
8	02 Dec	–	1222	725	717	1442	12.1
9	03 Dec	–	0	0	1367	1367	13.3
10	04 Dec	–	1222	824	0	824	22.0
11	05 Dec	2448	0	0	0	0	35.2
12	06 Dec	1998	1974	1530	583	2113	1.4
13	07 Dec	2338	0	0	1308	1308	14.3
14	08 Dec	2117	0	0	820	820	22.1
**Mean**		**2225**	**571**	**383**	**550**	**933**	**20.3**
**P3: EI used 2900 kcal·day^{-1}; FFM 57.5 kg; required EEE 1750 kcal·day^{-1}**							
1	08 Dec	–	2680	1722	0	1722	20.5
2	09 Dec	–	2680	0	0	0	50.4
3	10 Dec	–	0	2492	0	2492	7.1
4	11 Dec	–	0	0	0	0	50.4
5	12 Dec	–	864	894	0	894	34.9
6	13 Dec	–	2680	0	0	0	50.4
7	14 Dec	–	2680	3374	0	3374	-8.2
8	15 Dec	–	2680	2722	0	2722	3.1
9	16 Dec	–	2680	0	0	0	50.4
10	17 Dec	–	0	918	0	918	34.5
11	18 Dec	2560	0	1807	0	1807	19.0

12	19 Dec	2744	864	2493	0	2493	7.1
13	20 Dec	2992	2680	766	0	766	37.1
14	21 Dec	3157	2680	2236	0	2236	11.5
**Mean**		**2863**	**1655**	**1387**	**0**	**1387**	**26.3**

EA = energy availability; EEE = exercise energy expenditure; EI = energy intake; FFM = fat-free mass. EI used is the habitual intake (mean of Days 2–5) applied as a constant in the calculation of EA on every day; recorded EI is the intake measured with the image-based food record on Days 11 to 14 (study Days 19 to 22), shown for comparison and not used in the calculation of EA; – = not recorded. FFM is the DXA-derived value at T1. Required EEE is the daily expenditure needed to reach the prescribed target of 20 kcal·kg FFM^{-1}·day^{-1}, calculated as habitual EI minus the product of the target and DXA-derived FFM, and is therefore constant within a participant. Prescribed cycling EEE is the expenditure predicted from the time prescribed in each training zone and the participant-specific gross mechanical efficiency. Completed EEE was taken from the training files uploaded to the athlete-monitoring platform; other-modality EEE comprises running, swimming and other recorded activity. Achieved gross EA was calculated as (EI − total completed EEE)/FFM using the constant habitual EI. Mean completed EEE reached 97%, 98% and 79% of the required value for P1, P2 and P3, respectively.
